# Immune Checkpoint Inhibition as a Novel Strategy for Microsatellite Instability-High Duodenal Adenocarcinoma: A Report of Three Cases

**DOI:** 10.70352/scrj.cr.25-0645

**Published:** 2025-12-18

**Authors:** Kojiro Shirabe, Tetsuro Kawazoe, Tomoya Harima, Sho Nambara, Yasuo Tsuda, Tomonori Nakanoko, Koji Ando, Eiji Oki, Tomoharu Yoshizumi

**Affiliations:** 1 Department of Surgery and Science, Graduate School of Medical Sciences, Kyushu University, Fukuoka, Fukuoka, Japan; 2 Department of Advanced Medicine and Innovative Technology, Kyushu University Hospital, Fukuoka, Fukuoka, Japan

**Keywords:** duodenal adenocarcinoma, microsatellite instability, immune checkpoint inhibitor

## Abstract

**INTRODUCTION:**

Duodenal adenocarcinoma (DA) is an extremely rare malignancy, accounting for less than 1% of all gastrointestinal cancers. Most cases are diagnosed at an advanced stage, making curative resection difficult and leading to a poor prognosis. Recent advances in tumor immunology have identified microsatellite instability-high (MSI-H) and deficient mismatch repair (dMMR) as predictive biomarkers for immune checkpoint inhibitors (ICIs). However, clinical evidence regarding their efficacy in DA remains limited. We report 3 cases of MSI-H/dMMR DA.

**CASE PRESENTATION:**

(a) Case 1: A 55-year-old woman underwent pancreaticoduodenectomy for DA and was subsequently diagnosed with Lynch syndrome. She has remained recurrence-free for 5 years and 2 months postoperatively. (b) Case 2: A 75-year-old man with locally advanced DA and impaired oral intake declined pancreaticoduodenectomy. After gastrojejunostomy, pembrolizumab was administered, achieving durable disease control for 1 year and 10 months. (c) Case 3: A 65-year-old woman with DA invading adjacent organs received neoadjuvant pembrolizumab. Following bowel obstruction, she underwent partial small intestine resection, and pathological examination revealed a complete response. She has remained recurrence-free for 3 years and 2 months.

**CONCLUSIONS:**

Surgical resection remains the standard treatment for DA. However, in MSI-H/dMMR cases, ICIs are expected to be effective, as observed in other gastrointestinal cancers. Pembrolizumab may represent a useful neoadjuvant option, and in patients who achieve a pathological response, nonsurgical management could also be considered.

## Abbreviations

CA19-9
: carbohydrate antigen 19-9
CEA
: carcinoembryonic antigen
DA
: duodenal adenocarcinoma
dMMR
: deficient mismatch repair
EGD
: esophagogastroduodenoscopy
ICI
: immune checkpoint inhibitor
MSI-H
: microsatellite instability-high
NCD
: National Clinical Database
ORR
: overall response rate
PD-1
: programmed death-1
SUV_max_
: maximum standard uptake value

## INTRODUCTION

Duodenal adenocarcinoma (DA) is a rare malignancy, accounting for less than 1% of all gastrointestinal cancers.^1,2)^ Because of its rarity, prospective clinical trials are limited, and current therapeutic strategies are largely extrapolated from gastric or colorectal cancers. Endoscopic or surgical resection remains the standard treatment for resectable cases and is considered the only potentially curative option.^3)^ However, many patients are diagnosed at an advanced stage, making curative resection difficult and resulting in a poor prognosis.

Recent advances in tumor immunology have highlighted molecular subtypes such as microsatellite instability-high (MSI-H) and deficient mismatch repair (dMMR), which show marked sensitivity to immune checkpoint inhibitors (ICIs), particularly programmed death-1 (PD-1) blockade. Although MSI-H/dMMR has been identified in a subset of duodenal cancers, clinical evidence supporting the efficacy of ICIs in this setting remains scarce. While trials such as KEYNOTE-158 and ZEBRA have demonstrated the efficacy of pembrolizumab in small bowel adenocarcinoma, real-world data and case reports are still limited.^4,5)^

Herein, we report all 3 cases of MSI-H/dMMR DA that have been encountered at our institution. In 2 cases, pembrolizumab was administered either as neoadjuvant therapy or for disease control; notably, one patient achieved a pathological complete response following curative resection. These cases provide additional evidence supporting the potential role of ICIs in the multimodal management of MSI-H/dMMR DA.

## CASE PRESENTATION

### Case 1

**Fig. 1:**
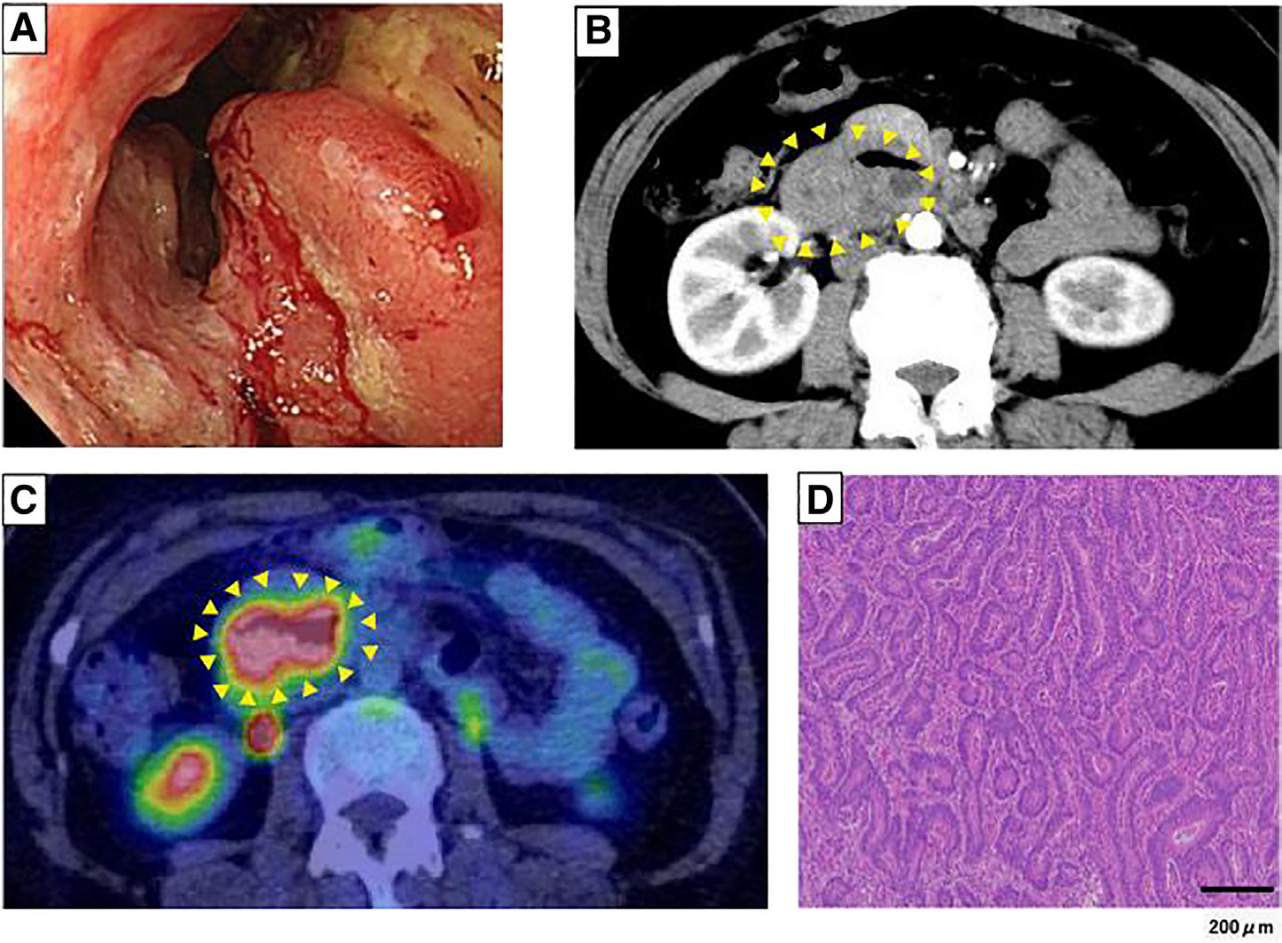
Esophagogastroduodenoscopy revealed an ulcerative lesion in the 3rd portion of the duodenum (**A**). CT showed duodenal wall thickening (arrowheads) without nodal or distant metastases (**B**). PET-CT demonstrated uptake in the descending duodenum (SUV_max_ 11.4) (arrowheads) without spread (**C**). Biopsy confirmed adenocarcinoma (**D**; scale bar: 200 μm). SUV_max_, maximum standard uptake value

A 55-year-old woman presented with fatigue and anemia. Esophagogastroduodenoscopy (EGD)^6)^ revealed duodenal cancer, and she was referred to our hospital. She had a strong family history of cancer: her father and sister had hepatocellular carcinoma, her mother had cholangiocarcinoma, and her brother had rectal cancer. Laboratory tests showed mild anemia (hemoglobin, 11.4 g/dL). Tumor marker levels were within normal limits (carcinoembryonic antigen [CEA], 1.1 ng/mL; carbohydrate antigen 19-9 [CA19-9], 3.0 U/mL). EGD revealed an ulcerative lesion in the third portion of the duodenum (**Fig. 1A**). CT demonstrated duodenal wall thickening without lymph node or distant metastases (**Fig. 1B**). PET-CT showed abnormal uptake in the descending duodenum (maximum standard uptake value [SUV_max_], 11.4) without evidence of nodal or distant spread (**Fig. 1C**). A biopsy confirmed adenocarcinoma (**Fig. 1D**). Standard pancreaticoduodenectomy was performed. A postoperative pancreatic fistula developed but improved with conservative management, and the patient was discharged on POD 33. Pathological diagnosis was pT3N0M0, Stage IIA, according to the 8th TNM classification.^7)^ Genetic testing revealed MSI-H with a pathogenic variant in *MSH2*. After genetic counseling, the patient was diagnosed with Lynch syndrome. She remains recurrence-free 5 years and 2 months after surgery.

### Case 2

**Fig. 2:**
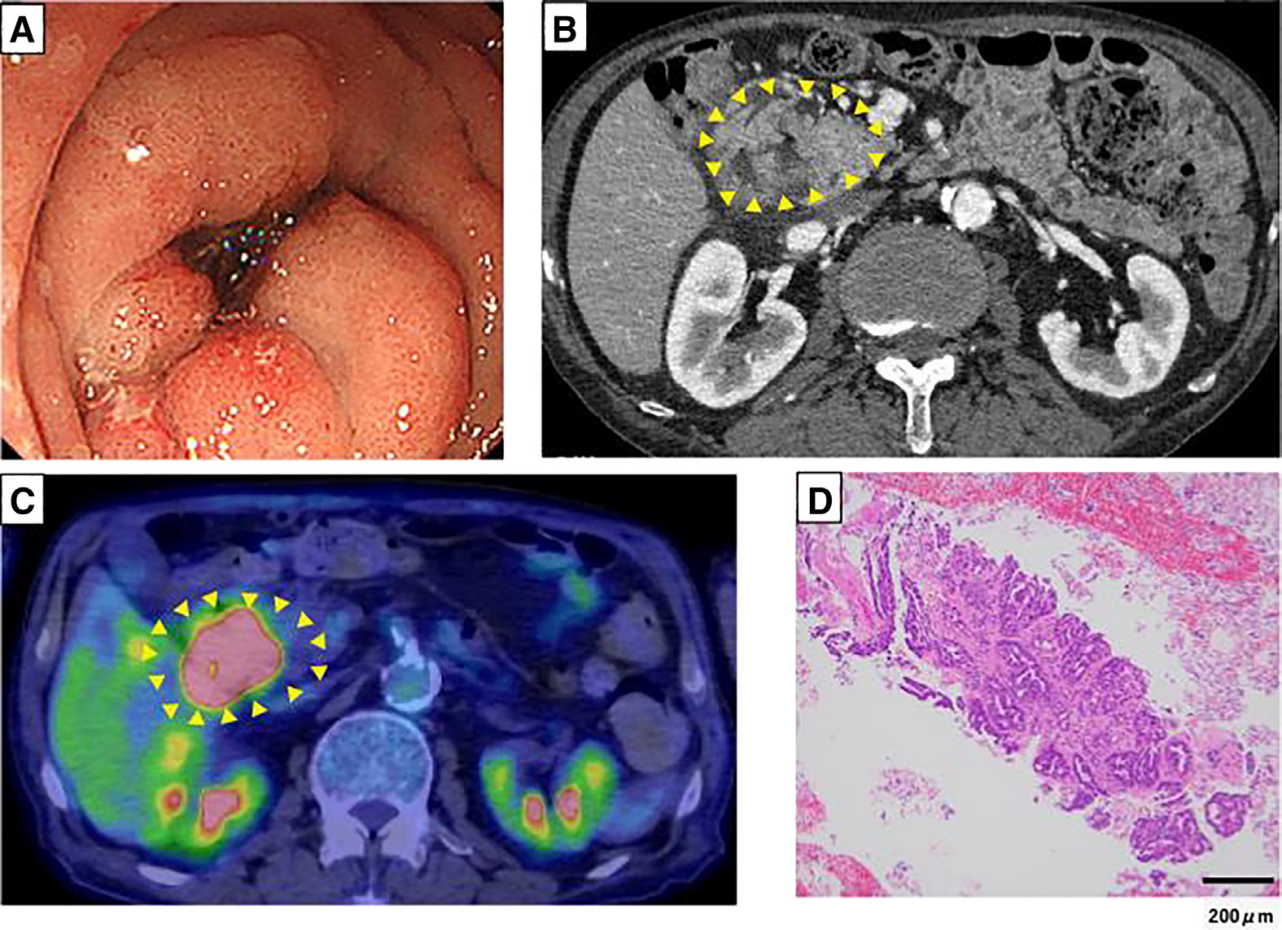
Esophagogastroduodenoscopy revealed circumferential thickening from the supraduodenal ampulla to the descending duodenum, obstructing passage (**A**). CT showed thickening with discontinuous wall, microperforation, and abscess (arrowheads) (**B**). PET-CT demonstrated uptake in the lesion (arrowheads) (SUV_max_ 19.6) (**C**). Biopsy confirmed adenocarcinoma (**D**; scale bar: 200 μm). SUV_max_, maximum standard uptake value

A 75-year-old man presented with weight loss and was referred for suspected duodenal cancer after a CT revealed a 3.5-cm mass in the pancreatic head. At referral, oral intake was severely impaired. Laboratory tests showed elevated CEA (5.9 ng/mL), while CA19-9 was within normal limits (7.2 U/mL). EGD revealed circumferential wall thickening from the supraduodenal ampulla to the descending duodenum, obstructing passage of the scope (**Fig. 2A**). Endoscopic ultrasonography demonstrated a poorly demarcated, hypoechoic mass in the uncinate process of the pancreas, raising suspicion of pancreatic cancer. CT revealed wall thickening from the descending duodenum to the inferior duodenal angle with discontinuous wall, microperforation, and abscess formation (**Fig. 2B**). PET-CT showed abnormal uptake in the lesion (SUV_max_, 19.6) (**Fig. 2C**). A biopsy confirmed adenocarcinoma (**Fig. 2D**). Genetic testing revealed MSI-H and HER2 negativity.

Because the disease was locally advanced, pancreaticoduodenectomy was recommended; however, the patient declined surgery for personal reasons. Gastrojejunostomy was performed to restore oral intake, followed by pembrolizumab therapy. Tumor shrinkage was achieved, and stable disease was maintained for 22 months.

### Case 3

**Fig. 3:**
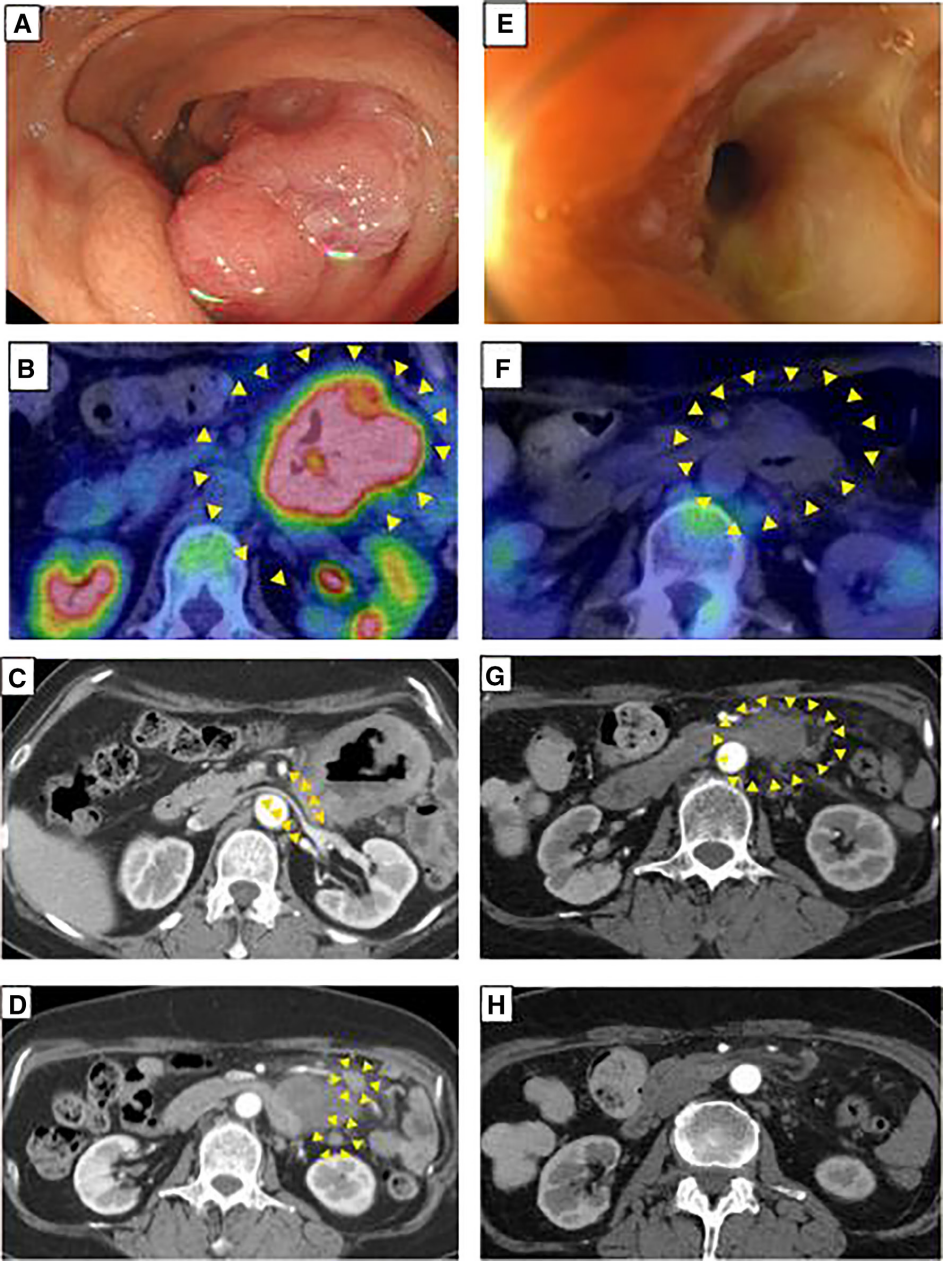
Esophagogastroduodenoscopy revealed a circumferential Borrmann type 2 lesion, 5–6 cm in size, in the horizontal part of the duodenum (**A**). PET-CT demonstrated abnormal uptake in the horizontal portion with an SUV_max_ of 12.7 (arrowheads) (**B**). CT showed wall thickening from the 3rd to 4th portion of the duodenum with suspected invasion into adjacent organs including the renal vein (arrowheads) (**C**); and multiple enlarged mesenteric lymph nodes were observed, suggesting metastasis (arrowheads) (**D**). Esophagogastroduodenoscopy showed tumor shrinkage with persistent stenosis (**E**); PET-CT demonstrated disappearance of abnormal uptake (arrowheads) (**F**); CT revealed the primary lesion was reduced in size (arrowheads) (**G**); and resolution of mesenteric lymph nodes (**H**). SUV_max_, maximum standard uptake value

A 65-year-old woman presented with abdominal pressure and discomfort. Endoscopy performed at another hospital revealed duodenal cancer, and she was referred to our institution. Laboratory results showed anemia (hemoglobin, 8.6 g/dL), while tumor markers (CEA and CA19-9) were within normal limits. EGD revealed a circumferential type 2 lesion measuring 5–6 cm in the horizontal part of the duodenum (**Fig. 3A**). CT demonstrated wall thickening in the 3rd and 4th portions of the duodenum, causing displacement of adjacent structures, including the renal vein (**Fig. 3B**). Biopsy confirmed adenocarcinoma, and genetic testing revealed MSI-H.

**Fig. 4:**
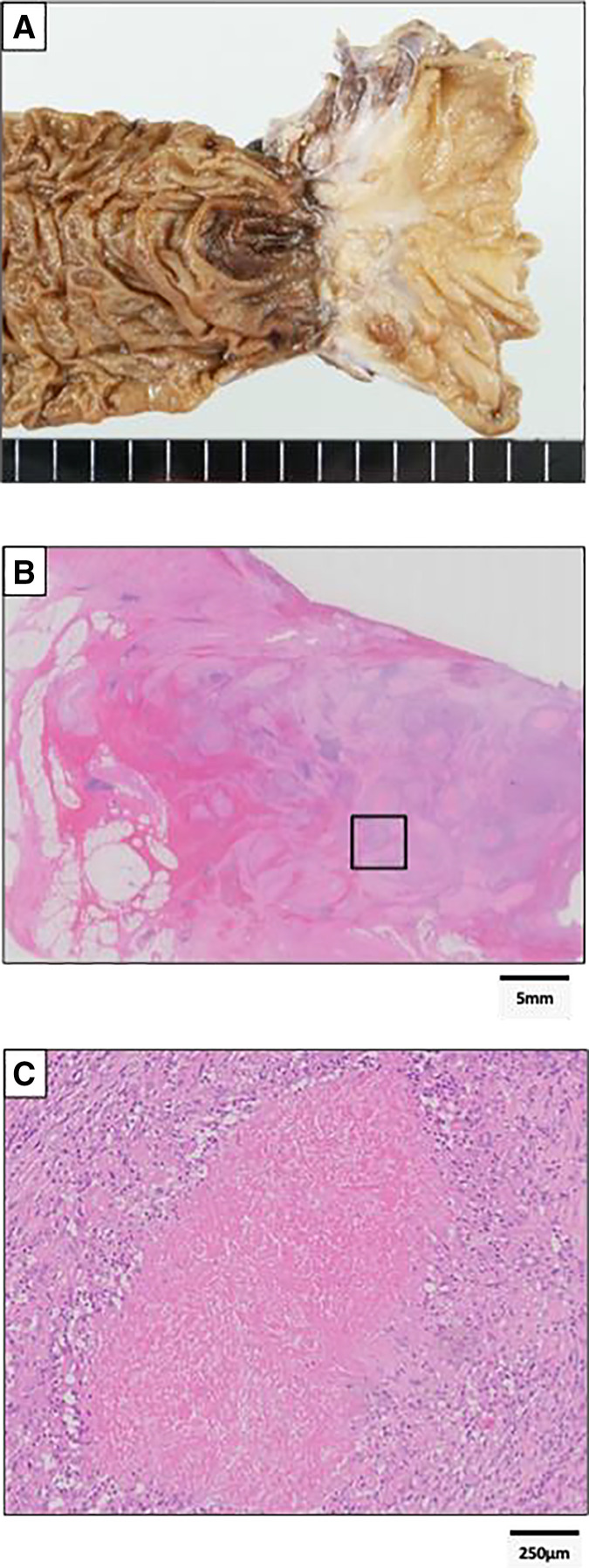
The tumor had shrunk and become scarred, resulting in duodenal stenosis (**A**). Pathological findings showed massive necrosis with granulomatous reaction, accompanied by fibrosis, lymphocytic aggregates, infiltration of foamy macrophages, focal hyalinization, and calcification (**B**, **C**).

Given the locally advanced disease, surgical resection with curative intent was considered; however, because of adjacent organ invasion and MSI-H status, neoadjuvant pembrolizumab was initiated. After 2 courses, the patient developed vomiting, and CT demonstrated bowel obstruction due to duodenal stenosis. EGD showed tumor shrinkage but persistent stenosis (**Fig. 3E**). From an oncologic standpoint, pancreaticoduodenectomy with lymph node dissection would have been preferable. However, the patient declined this procedure due to concerns about postoperative functional impairment. In addition, current guidelines note that there is no evidence that lymph node dissection improves survival outcomes. For these reasons, laparoscopic partial distal duodenal resection was performed. The postoperative course was uneventful, and the patient was discharged on POD 13. Postoperative pathological examination revealed no residual tumor or nodal metastases. The patient had experienced bowel obstruction preoperatively, which was caused by scarring resulting from tumor shrinkage (**Fig. 4A**). Pathological findings showed massive necrosis with granulomatous reaction, accompanied by fibrosis, lymphocytic aggregates, infiltration of foamy macrophages, focal hyalinization, and calcification (**Fig. 4B** and **4C**). A pathogenic variant in MSH2 was identified, and after genetic counseling, the patient was diagnosed with Lynch syndrome. No adjuvant therapy was administered, and the patient has remained recurrence-free for 3 years and 2 months after surgery.

## DISCUSSION

Duodenal cancer is rare, with an estimated incidence of 3.7 per million in North America^8)^ and 2.9–4.3 per million in Western countries.^9,10)^ According to the Japanese National Clinical Database (NCD), the incidence is higher in Japan, at 23.7 cases per million.^11)^ A nationwide Japanese survey reported that 5.6% of duodenal cancers were MSI-H, ranking 4th among solid tumors after endometrial (16.9%), small bowel (8.6%), and gastric cancers (6.7%).^12)^ Duodenal cancer is often diagnosed at advanced stages, and the prognosis remains poor, with 5-year survival rates ranging from 10% to 75% for locally advanced disease and only 4.7% for metastatic cases.^13–15)^

Surgery remains the standard treatment for resectable cases, with pancreaticoduodenectomy being the preferred approach. Lymph node metastasis has been identified as a poor prognostic factor; however, the optimal extent of lymph node dissection has not been established. For distal duodenal cancer, pancreaticoduodenectomy may be excessively invasive, and segmental resection is sometimes performed. Because this may result in insufficient lymph node dissection, its curative potential remains controversial.^1)^

de Bakker et al. reported that, among 74 patients who underwent surgical resection for DA between 2000 and 2019, 16 (21.6%) were MSI-H—a significantly higher frequency than in other gastrointestinal cancers and higher than that reported in Japan.^16,17)^

ICIs have improved survival across multiple solid tumors, including gastrointestinal cancers. In the KEYNOTE-158 trial, pembrolizumab achieved an overall response rate (ORR) of 30.8% in previously treated non-colorectal solid tumors and a notably higher ORR of 48.0% in small bowel cancer.^4)^ Similarly, the GARNET trial demonstrated the efficacy of dostarlimab in previously treated dMMR solid tumors (n = 106), reporting an ORR of 38.7% and a complete response rate of 7.5%. Notably, small bowel cancers achieved an ORR of 43.3%, higher than most other tumor types.^18)^ The ZEBRA trial also reported the efficacy of pembrolizumab in previously treated advanced small bowel cancers, including duodenal cancer.^5)^ Because these trials evaluated the efficacy of ICIs in second-line or later settings, the response rates were relatively limited. However, a recent report from the CheckMate 8HW trial showed that progression-free survival was prolonged when ICIs were used as the first-line therapy, suggesting that even more favorable outcomes may be expected in the future.^19)^

Although evidence remains limited due to the rarity of duodenal cancer, accumulating reports support the effectiveness of pembrolizumab in dMMR/MSI-H cases.^20)^ To date, including ours, 9 cases of MSI-H duodenal cancer treated with ICIs have been reported (**Table 1**).^20–23)^ These findings suggest that pembrolizumab offers superior safety and efficacy compared with conventional chemotherapy in MSI-H duodenal cancer. However, several points of caution should be noted regarding these reports. First, these reports are retrospective case studies, and the possibility of publication bias cannot be excluded. Furthermore, to our knowledge, the perioperative use of pembrolizumab for DA has not been established. While perioperative or neoadjuvant administration of pembrolizumab has demonstrated efficacy in several malignancies, including melanoma and non-small-cell lung cancer, its role in the perioperative management of MSI-H DA remains unreported.^24,25)^

**Table 1:** Reported cases of MSI-high duodenal cancer^20–23)^

No.	Age	Sex	Primary site	Symptoms at presentation	Histology	Somatic mutation in MMR genes	cTumor stage	Lynch syndrome	Treatment	Period since diagnosis (months)	Clinical outcome	Studies
3	65	F	Third portion of the duodenum	Fatigue	Well to moderately differentiated adenocarcinoma	MSH2 loss	cT3N0M0 cStage IIA	Yes	Surgery	62	Alive	Present case
2	75	M	Duodenal bulb to the 2nd portion	Weight loss	Adenocarcinoma		cT3N2M0 cStage IIIB	No	Unresectable/ recurrent disease Pembrolizumab PR	22	Alive	Present case
1	55	F	Third portion of the duodenum	Abdominal pressure and discomfort	Well to moderately differentiated adenocarcinoma	MSH2 loss	cT3N2M0 cStage IIIB	Yes	Neoadjuvant Pembrolizumab cCR	38	Alive	Present case
4	54	M	—	Abdominal pain	Moderately differentiated adenocarcinoma	PMS2 and MLH1 loss	T4N+M0	No	Neoadjuvant Pembrolizumab pCR	55	Alive	Ziane Bouziane et al.^20)^
5	76	M	—	Weight loss	Well-differentiated adenocarcinoma	PMS2 loss	T4N+M0	No	Neoadjuvant Pembrolizumab pCR	53	Alive	Ziane Bouziane et al.^20)^
6	53	F	—	Anemia	Moderately differentiated adenocarcinoma	PMS2 loss	T4N+M0	No	Neoadjuvant Pembrolizumab pCR	116	Alive	Ziane Bouziane et al.^20)^
7	50	M	Duodenal bulb	Abdominal pain	Poorly differentiated Medullary carcinoma	MLH1 and PMS2 loss	cT4N2M1 cStage IV	Yes	Unresectable/ recurrent disease Pembrolizumab PR	—	Alive	Liu et al.^21)^
8	55	F	Descending duodenum	Abdominal discomfort	Poorly differentiated adenocarcinoma	MLH1 loss	cT3N1M0 cStage IIIA	No	Unresectable/ recurrent disease Pembrolizumab cCR	63	Alive	Kong et al.^22)^
9	73	F	Duodenal bulb	Abdominal pain	High-grade intraepithelial neoplasia	PMS2 loss	cT3N2M0 cStage IIIB	No	Neoadjuvant Pembrolizumab PR	22	Alive	Wang et al.^23)^

F, female; M, male; MMR, mismatch repair; MSI, microsatellite instability; pCR, pathological complete response; PR, partial response

In addition to these considerations, according to the Japanese clinical practice guidelines for duodenal cancer management, fluoropyrimidine plus oxaliplatin is recommended as the first-line chemotherapy for unresectable duodenal cancer. However, based on these previous reports, we decided to administer pembrolizumab as the first-line treatment.

In our series, 1 patient underwent pancreaticoduodenectomy as standard care, while 2 patients received pembrolizumab-based therapy—one as palliative treatment following bypass surgery and the other as neoadjuvant therapy, achieving a pathological complete response. In rectal cancer, PD-1 inhibitors are being investigated as a strategy to avoid surgery.^26)^ Given that surgery for duodenal cancer is particularly invasive,^27)^ treatment with ICIs may provide a potential alternative in MSI-H cases, provided careful monitoring and informed consent are ensured. Notably, one of our patients underwent pancreaticoduodenectomy before MSI-H status was identified. Had the diagnosis been made preoperatively, neoadjuvant therapy could have been considered, even in a resectable case. Considering the relatively high frequency of MSI-H in duodenal cancer, we propose that MSI testing should be performed preoperatively in all patients.

Two of our patients were ultimately diagnosed with Lynch syndrome based on genetic testing and counseling. Lynch syndrome is an autosomal dominant disorder associated with a markedly increased risk of small bowel cancer (~4%), nearly 100-fold higher than in the general population.^28)^ Mutations in mismatch repair genes, particularly *MLH1*, *MSH2*, and *MSH6*, are associated with increased risks of gastric and duodenal cancers. Specifically, *MSH2* mutations have been linked to small bowel cancer, although the underlying mechanism remains unclear.^29)^ ICIs are highly effective in Lynch syndrome-associated colorectal cancers,^30)^ and similar benefits may extend to duodenal cancer, although further studies are needed.

In our series, 1 patient treated surgically remained recurrence-free, while 2 patients treated with systemic therapy achieved durable disease control, including one complete pathological response. These findings suggest that pembrolizumab monotherapy may represent a viable option for selected patients with MSI-H duodenal cancer, raising the possibility that nonoperative management could be considered in the future.

## CONCLUSIONS

Our case series demonstrates that pembrolizumab can achieve durable disease control and even a pathological complete response in MSI-H DA, including in patients with Lynch syndrome. While surgical resection remains the standard curative treatment, these findings highlight the potential role of ICIs as neoadjuvant, palliative, or alternative therapeutic strategies in selected patients. Further accumulation of clinical cases and prospective studies is warranted to establish optimal treatment approaches and to clarify the role of immunotherapy in the multimodal management of MSI-H DA.

## DECLARATIONS

### Availability of data and materials

The datasets generated and analyzed during the current study are available from the corresponding author upon reasonable request.
